# Wireless Fractal Ultra-Dense Cellular Networks

**DOI:** 10.3390/s17040841

**Published:** 2017-04-12

**Authors:** Yixue Hao, Min Chen, Long Hu, Jeungeun Song, Mojca Volk, Iztok Humar

**Affiliations:** 1 School of Computer Science and Technology, Huazhong University of Science and Technology, Wuhan 430074, China; yixuehao@hust.edu.cn (Y.H.); longhucs@hust.edu.cn (L.H.); jsong@hust.edu.cn (J.S.); 2 Laboratory for Telecommunications, Faculty of Electrical Engineering, University of Ljubljana, Trzaska 25, SI-1000 Ljubljan, Slovenia; mojca.volk@fe.uni-lj.si (M.V.); iztok.humar@fe.uni-lj.si (I.H.)

**Keywords:** 5G, ultra-dense small cell network, coverage probability

## Abstract

With the ever-growing number of mobile devices, there is an explosive expansion in mobile data services. This represents a challenge for the traditional cellular network architecture to cope with the massive wireless traffic generated by mobile media applications. To meet this challenge, research is currently focused on the introduction of a small cell base station (BS) due to its low transmit power consumption and flexibility of deployment. However, due to a complex deployment environment and low transmit power of small cell BSs, the coverage boundary of small cell BSs will not have a traditional regular shape. Therefore, in this paper, we discuss the coverage boundary of an ultra-dense small cell network and give its main features: aeolotropy of path loss fading and fractal coverage boundary. Simple performance analysis is given, including coverage probability and transmission rate, etc., based on stochastic geometry theory and fractal theory. Finally, we present an application scene and discuss challenges in the ultra-dense small cell network.

## 1. Introduction

With the rapid popularization of mobile internet, an explosive expansion in the amount of mobile terminals with access to a wireless network can be observed. It is predicted that the total number of devices with access to a wireless network in the world will reach 75 billion in 2020, and monthly mobile traffic will exceed 24.3 exabytes [1]. Therefore, the explosive increase in mobile data services makes it hard for the traditional cellular network architecture to cope with the massive wireless traffic and with users’ communication quality requirements, which is also one of challenges to be coped with in future 5G communication technology [2]. To meet this challenge, some researchers are focusing on adaptive and scalable heterogeneous communication frameworks; e.g., [3,4,5]. Besides, current research is widely concerned with small cell base stations (BSs) due to their low transmit power consumption and flexible deployment capabilities [6,7,8] . An ultra-dense deployment of small cell BSs has the capability to provide higher transmission rates and more reliable wireless connection, thus meeting the demands of the newly introduced massive wireless traffic.

With the ultra-dense deployment of small cell BSs, the coverage radius of a small cell is shrinking. This causes a considerable increase in the frequency of handoffs performed for mobile users, which results in an increasing attention of the coverage boundary of small cell BSs [9]. Until recently, the coverage boundary of wireless cellular networks has been comprehensively discussed. In a 3G cellular network, the requirements on wireless traffic are relatively small. Thus, we can increase the density of the macrocell BSs deployment to improve the transmission rate and meet user requirements on communication quality. In this type of network, the BSs density is low and the transmit power is high. Therefore, when BSs are uniformly distributed, their coverage boundary can be described as a regular hexagon. In a 4G cellular network (e.g., a long-term evolution (LTE) network), with increasing requirements on wireless traffic, the transmission rate can be improved through deployment of microcells (e.g., femtocell BSs) [10] . As the density of the BSs increases, the coverage boundary based on a regular hexagon no longer applies because its relevant assumption is too ideal. Based on measurements of real data from BSs, the locations of BSs can be modeled as independent Poisson point processes (PPPs) [11]. Using the Poisson–Voronoi tessellation (PVT) method, it can be concluded that the coverage boundary of a 4G cellular network is an irregular polygon shape.

In a 5G cellular network, in order to meet further increasing requirements for wireless traffic, the transmission rate of the network can be improved through an ultra-dense deployment of small cell BSs. Since small cell BSs are deployed in urban areas, the irregularity in the geometric distribution of buildings and blocking objects produce absorption, reflection, scattering, and diffraction to electromagnetic waves at different levels of intensity [12]. Therefore, these signals are subjected to the aeolotropy path loss fading, shadow fading, and multi-path fading during radiation propagation of wireless signals transmission, which causes an increasingly complex electromagnetic fading environment and coverage boundary situation. Ge et al. in [13] conducted actual measurements for an ultra-dense small cell network. It was concluded from the results that the coverage boundary of an ultra-dense small cell network bears stronger burstiness and irregularity compared to a traditional coverage boundary of a cellular network, and it can be described by Pareto distribution. In conclusion, macrocells and microcells are deployed in irregular polygon-shaped cell networks, and their density is about 8–10 $BSs/km^{2}$. For wireless fractal ultra-dense small cell networks, macrocells and ultra-dense small cells are deployed, and their density is about 40–50 $BSs/km^{2}$. Furthermore, the interference and coverage redundancy of ultra-dense small cell networks are much higher than the irregular polygon-shape cell networks. In Table 1, we compare various performance parameters for the coverage boundary of 3G, 4G, and 5G networks.

In this paper, a wireless fractal ultra-dense cellular network and its two main features are discussed in Section 2. We propose a simple performance analysis (including coverage probability and transmission rate, etc.) for wireless fractal ultra-dense cellular networks based on stochastic geometry theory and the fractal theory in Section 3, and discuss the application scenes and challenges in Section 4. Finally, Section 5 concludes this paper.

## 2. Wireless Fractal Ultra-Dense Small Cell Networks

### 2.1. Evolution of Wireless Cellular Coverage Boundary

In a 3G cellular network, when macrocell BSs are deployed evenly with equal distance, the wireless cellular coverage shape is a regular hexagon. As the density of BSs in a 4G cellular network is increased, the regular hexagon coverage model largely deviates from the space deployment of actual BSs [14] . In recent years, with the development of the stochastic geometry theory for wireless communication networks, Jeffrey et al. [15] firstly conducted modeling for the distribution of BSs in a cellular network with the homogeneous PPP. He deduced a simple theoretical expression for user-level performance such as coverage probability and transmission rate, and made a comparison with the regular hexagon cellular network model and the actual cellular system to verify the rationality of the PPP model. The advantage of the PPP model is that points are distributed independently and randomly. Furthermore, a simple and skillful performance expression can be given with the PPP model. Based on the PPP model and PVT method, we can obtain that the coverage boundary of a 4G cellular network is an irregular polygon.

**Figure 1:**
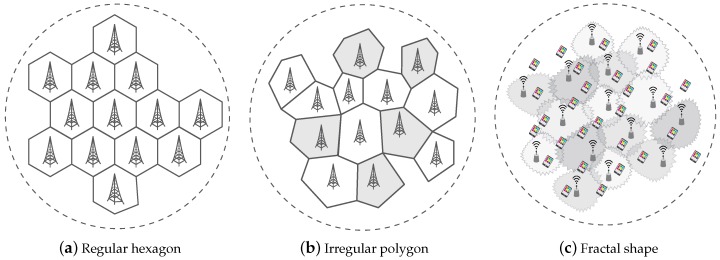
The evolution of wireless cellular coverage boundaries.

However, regarding the ultra-dense deployment of small cell BSs in a 5G network, modeling for all BSs into a PPP model does not correspond to an actual system. This is because the coverage radius is shrinking as the deployment density of small cell BSs increases, which makes user handoff among different cells increasingly frequent. Thus, the user is increasingly sensitive to the coverage boundary of small cell BSs. Furthermore, the influence of the communication environment on the cellular network coverage boundary is not taken into consideration. Based on further research outcomes reported in [13], the coverage boundary for ultra-dense small cell BSs takes on a fractal feature. The evolution of wireless cellular coverage boundaries is shown in Figure 1.

### 2.2. Main Features of Fractal Ultra-Dense Small Cell Networks

In accordance with the above discussion, there are two main features for fractal ultra-dense small cell BSs: (i) the path loss fading; (ii) the fractal nature of coverage boundary. We first introduce the anisotropism of path loss fading. In a 3G cellular network, uniform path loss exponents are adopted in a path loss model to evaluate the path loss in the whole cellular network, and discrimination analysis for the path loss is not conducted on different transmission paths. This assumption is obviously not adapted to 4G or 5G networks—especially not to 5G cellular networks after introducing an ultra-dense deployment of small cell BSs. Therefore, some studies consider path attenuation with different parameters with different distances, and put forth two main path loss models: the first is the non-line-of-sight (NLOS) model and the line-of-sight (LOS) path loss model [16]; the second is the multi-slope path loss model [17]. However, these models only take into consideration the situation where the different distances between user and BS cause different path fading, whereas the anisotropism of signal attenuation (i.e., the difference in path attenuation when the distance between the user and the BS is equal but the direction of the user is different) is not taken into consideration. Therefore, in this paper, let *r* denote the distance between the user *u* and the small cell BS *s*, and α denote path loss exponent. Based on traditional standard path loss model, $l_{u,s}=r^{-\alpha }$, we assume that the path loss exponent α is different for different propagation paths. Based on the work in [13], we assume that the path loss exponent α follows a Gamma distribution, and then we can obtain the anisotropic path loss model as follows:

(1) $$l_{u,s}=r^{-\alpha },\;f\left(\alpha \right)=\frac{{\left(\alpha -2\right)}^{\left(\tau -1\right)}\exp\left(\frac{-(\alpha -2)}{\theta }\right)}{\Gamma \left(\tau \right)\theta^{\tau }},\;\beta \geq 2$$

where τ is shape parameter, θ is scale parameter, and Γ(τ) is the Gamma, please confirm that your intended meaning is retained. function of parameter τ.

Next we will discuss the coverage boundary of small cell BSs. Typically, the probability density function on the border of small cell BSs bears a heavy tail feature [13], and that the distance *R* from a small cell BS to the coverage boundary of cellular networks bears a fractal feature (i.e., feature of self-similarity). This is because the signals from small cell BSs are subjected to different signal attenuation in different directions. Since the Pareto distribution can describe the space and the traffic of the self-similarity, we can obtain the probability density function (PDF) of the Pareto distribution as follows:

(2) $$f\left(R\right)=\left\{\begin{array}{cc} \frac{\varepsilon R^{-(\varepsilon +1)}}{R_{\min}^{-\varepsilon }-R_{\max}^{-\varepsilon }} & R_{\min}<R<R_{\max}\\ 0 & \mathrm{otherwise} \end{array}\right.$$

where ε is the parameter of fractal feature, $R_{\min}$ is the minimum value of *R*, and $R_{\max}$ is the maximum value of *R*. We can define the distance from users to the small cell BS as $r=\beta R_{max}$. Since the users are evenly distributed in the coverage of small cell BSs, the $\beta^{2}$ follow with even distribution on (0,1).

## 3. Performance of the Fractal Small Cell Networks

In accordance with the analysis in Section 2, it can be concluded that the coverage boundary of small cell BSs takes on a fractal feature after the ultra-dense deployment of small cell BSs, and that there is anisotropism in the fading path loss. Through the path loss model and the fractal model given in Section 2, we can obtain the signal to interference plus noise ratio (SINR) outage probability and the transmission rate of the ultra-dense small cell BSs, and we can thus obtain the energy efficiency of the ultra-dense small cell networks.

In this paper, we assume that the small cell BSs and the location of the user terminals follow with the homogeneous PPP $\Phi_{B}$ and $\Phi_{u}$ on a two-dimensional plane, and that the distribution density is $\lambda_{B}$ and $\lambda_{u}$, respectively. We define the probability that the received SINR at the user terminal is less than its desired target as $\theta_{t}$, then we can conclude that the SINR outage probability $P_{\mathrm{cov}}$ is:

(3) $$P_{\mathrm{cov}}=Pr\left({\mathrm{SINR}}_{u}\leq \theta_{t}\right)=Pr\left(\frac{p_{s}l_{u,s}h_{u,s}}{I_{u}+\sigma^{2}}\leq \theta_{t}\right)$$

where $p_{s}$ and $\sigma^{2}$ are the transmit power of the serving small cell BS *s* and the power of noise, respectively, $h_{u,s}$ is the channel fading from serving the small BS *s* to the user terminal *u*, and $I_{u}$ is the aggregate interference at user, which is defined as follows [18]:

(4) $$I_{u}=\sum_{y\in \Phi_{I}\setminus u}p_{y}l_{u,y}h_{u,y}.$$

Substituting (1), (2), and (4) into (3), based on the Laplace transform and Poisson point process, we can obtain the approximate expression of the SINR outage probability. On the basis of the SINR outage probability, it can be concluded that the transmission rate for the user *u* is $r=\log_{2}\left(1+{\mathrm{SINR}}_{u}\right)$. As per transmission rate of the user, energy efficiency (EE) can be obtained as follows:

(5) $$EE=\frac{r}{\eta p_{s}+p_{c}}$$

where η is the efficiency of the transmit power amplifier and $p_{c}$ is the fixed static power consumption. From the user’s point of view, SINR outage probability is an important metric. The EE is an important measure for both users and network operators. This is because the EE not only considers the transmission rate, but also considers the energy consumption of a small cell BS.

## 4. Application Scenarios and Challenges

In this section, we discuss the application scenarios and challenges of wireless fractal ultra-dense cellular networks.

### 4.1. Application Scenarios

Since a small cell BS is characterised with low transmit power, small volume, and flexible deployment, it is mainly applied in two circumstances: (i) In areas where deployment of a macro cell BS is not convenient, such as in a residential community. Application of small cell BSs can reduce the coverage hole, improving communication and signal quality for users located on the border; (ii) In business districts and hot spots, such as shopping malls. As network traffic in these areas is relatively large, small cell BSs can enhance transmission capacity in these areas and reduce network traffic during rush hours.

Furthermore, in terms of wireless body sensor networks (BSN), with the development of wearable technology, more and more users wear sensing devices for health monitoring. For example, Chen et al. [19,20] designed a sustainable healthcare system based on smart clothing. Iyengar et al. [21] defined a framework that manages common tasks for healthcare. Fortino et al. [22] designed an open-source programming framework to support rapid and flexible prototyping and management of BSN applications. Moreover, Chen et al. [23] proposed an emotion communication system for psychological health. However, these works do not take the low transmit power of wireless body sensors into account, and these sensors are easily influenced by the environment. Thus, the signals of these sensors appear as a fractal phenomenon. For example, Ibaida et al. [24] discussed fractal-based ECG compression in wireless body sensor networks.

### 4.2. Challenges

As an ultra-dense deployment of small cell BSs causes fractal network coverage boundary and anisotropism of the path loss fading, wireless fractal ultra-dense small cell networks will be faced with the following challenges:

- *Controllability of small cell BSs:* With the development of software-defined network (SDN) technology, we can adopt SDN to separate the functions of a small cell BS and to make it only with forwarding function. When the traffic of a cellular network increases sharply (e.g., at large gatherings), the fractal coverage of a community may be realized through adjusting the transmit power of the small cell BS, thus alleviating the burden of traffic.
- *Cooperative communication of small cell BSs:* Through the discussion above, it can be concluded that the coverage boundary of wireless fractal ultra-dense cellular networks takes on fractal feature. Thus, cooperative communication between small cell BSs is not determined through traditional mutual distance between cells, but through the fractal feature of the border between neighboring cells; therefore, the relationship between user and cooperative cell is still a challenging problem.
- *Caching scheme deployment for small cell BSs:* In addition to communication, content caching may also be conducted by small cell BSs to decrease the traffic of a cellular network in rush hours. The existing caching schemes are all based on the fact that the coverage boundary of a cellular network is an irregular polygon. When the coverage boundary of a cellular network is fractal, how to conduct deployment for caching content and to increase cache hit ratio of files as per coverage of small cell BS is a challenging problem.

## 5. Conclusions

Ultra-dense deployments of small cell BSs is a key technology in the 5G cellular network architecture. This is because with ultra-dense small cell BSs, the ever-increasing traffic volumes generated by end users can be successfully handled. Furthermore, due to the low transmit power and flexible deployment of small cell BSs, energy consumption optimization of cellular deployment can be realized. However, with the ultra-dense deployment of small cell BSs, the user handoff between different small cell BSs is more and more frequent, and the user is more and more sensitive to the coverage boundary of small cell BSs. Therefore, in this paper, we discussed the coverage boundary of ultra-dense small cell BSs and gave the main coverage features for the ultra-dense small cell BSs: anisotropism of path loss fading and fractal coverage boundary. Furthermore, we analysed coverage probability, transmission rate, and EE of ultra-dense small cell networks. Finally, we discussed possible application scenes and challenges in fractal ultra-dense small cell networks.

## Figures and Tables

**Table 1:** Comparison of 3G, 4G, and 5G cellular networks. BS: Base station.

Cellular Network	Third Generation	Fourth Generation	Fifth Generation
Coverage Feature Deployment	Regular hexagon Macrocells BS	Irregular polygon Macrocells and microcell	Statistical fractal shape Macrocells and Ultra-dense small cells
BS density	Low (4–5 BSs/km${}^{2}$)	Middle (8–10 BSs/km${}^{2}$)	High (40–50 BSs/km${}^{2}$)
Transmit Power of Macrocell	High	High	High
Transmit Power of Small cell	N/A	N/A	Low
Interference	Low	Middle	High
Coverage Redundancy	low	Middle	High
Wireless Fractal Phenomenon	No	No	Yes
